# Language Learning for People Living with Dementia and Their Caregivers: Feasibility and the Quality of Experience

**DOI:** 10.3390/healthcare12070717

**Published:** 2024-03-25

**Authors:** Mariana Vega-Mendoza, Robbie S. Norval, Brittany Blankinship, Thomas H. Bak

**Affiliations:** 1 Psychology, Department of Health, Education and Technology, Luleå University of Technology, 971 87 Luleå, Sweden; 2 Department of Psychology, School of Philosophy, Psychology and Language Sciences, The University of Edinburgh, Edinburgh EH8 9JZ, UK; b.blankinship@ed.ac.uk (B.B.); thomas.bak@ed.ac.uk (T.H.B.); 3 Lingo Flamingo, Glasgow G41 3LP, UK; robbie.norval@lingoflamingo.co.uk; 4 Usher Institute, The University of Edinburgh, Edinburgh EH8 9AG, UK

**Keywords:** people living with dementia, family carers, language learning, non-pharmacological interventions, qualitative study

## Abstract

Background: A body of research from around the world has reported positive effects of bilingualism on cognitive ageing and dementia. However, little is known about whether foreign language learning could be applied as an intervention for people already living with dementia. Yet, before it is possible to determine the efficacy of language courses as an intervention for people living with dementia (PLWD), it is necessary to establish whether such an intervention is feasible. Our study explored this possibility. Methods: We conducted an exploratory study to examine the feasibility and tolerability of 2-week Italian beginner courses for PLWD in early stages and their family carers in two Scottish Dementia Resource Centres (DRCs). The courses were delivered by trained tutors from Lingo Flamingo, a social enterprise specialising in language teaching for older learners and learners with dementia. Twelve PLWD and seven carers participated in the study. Focus groups preceded and followed the courses. Additional post-course open interviews with the DRC managers were conducted, with a follow-up via telephone approximately one year later. Results: Qualitative content analysis resulted in 12 themes, 5 reflected in the interview schedule and 7 arising from the focus groups and interviews. Overall, the courses were perceived positively by PLWD, carers, and DRC managers, although a few logistically and linguistically challenging aspects were also mentioned. The courses were found to positively impact both the individual by increasing self-esteem and producing a sense of accomplishment as well as the group by creating a sense of community. Notably, no adverse effects (in particular no confusion or frustration) were reported. Conclusion: The positive outcomes of our study open a novel avenue for future research to explore foreign language training in dementia as an intervention and its implications.

## 1. Introduction

Dementia is characterised by progressive cognitive deficits [1] as well as by changes in mood [2] and behaviour [3]. The neuropsychiatric symptoms of dementia (NPSD), ranging from depression, low self-esteem, anxiety, apathy, and social withdrawal to irritability, agitation [4,5,6,7,8], and aggressiveness [9], have proven as difficult to address pharmacologically as the cognitive ones [10]. Majic et al. (2010) [9] assessed the prevalence of NPSD and their pharmacological treatment across 18 care homes in Berlin. Over 90% of patients exhibited NPSD, above all apathy, followed by agitation and depression. Neuroleptics and antidepressants were the most common pharmacological treatment. In a systematic review of randomised controlled trials to treat NPSD, Seitz et al. (2012) [11] concluded that the evidence supporting the efficacy of pharmacological treatments for NPSD in long-term care is limited.

The scarcity of effective pharmacological interventions for cognitive and neuropsychiatric manifestations of dementia has resulted in an increasing interest in potential non-pharmacological interventions [12,13,14] (see also [15] for a systematic review). In particular, leveraging protective lifestyle factors that have been suggested to delay the age of onset of dementia could prove to be an effective way to develop such interventions. One of these factors is engaging in mentally stimulating activities [16,17,18], and one of such activities could be learning of additional languages [19,20].

### 1.1. Effects of Bi/Multilingualism and Language Learning on Cognitive Ageing

Lifelong bilingualism (defined here as the ability to communicate in more than one language rather than a perfect, “native-like” command of all of them) has been associated with a later onset of dementia (e.g., [21,22,23]) and a better cognitive outcome in later life in healthy ageing ([24]; see also [25,26,27]). Importantly, these effects are not confined to lifelong bilingualism, and even a brief, one-week intensive language course can lead to a measurable improvement in attentional functions in participants from 18 to 78 years old; importantly, this benefit was preserved in those who continued practicing the language on average 5 h or more per week in a follow-up assessment nine months after the language course [28].

These effects extend to other brain pathologies; bilingualism has been associated with a better cognitive outcome after stroke [29] and a less severe presentation of post-stroke aphasia [30]. In terms of neuroimaging, bilingualism has been associated with increased white [31,32,33] and grey matter [34,35]; bilingual dementia patients are also able to maintain better cognitive functions than their monolingual counterparts with the same degree of cerebral atrophy [36]. However, given the large number of potentially relevant linguistic, psychological, and social variables and the complex interaction between them, it is not surprising that not all studies show the same results [37]. The exact effects of bilingualism on cognition are very complex and depend on the population from which the sample is drawn, the type of dementia [38], the type of tests [39], and a wide range of other interacting variables [40].

While the nature and extent of potential cognitive effects of bilingualism are influenced by a large number of interacting variables [40] and remain the focus of a heated scientific debate (for a review see [41]; see also [42,43]), language classes might have many benefits independently of their immediate cognitive effects; it has been proposed that learning new skills in older adulthood, such as learning a new language, can lead to enhanced quality of life, in particular, functional independence [44]. Moreover, a pilot study reported that learning a new language in healthy older adults aged 63–90 years old not only led to enhanced cognitive abilities (specifically, executive function), but also positive effects on self-confidence, autonomy, and overall well-being [45].

### 1.2. Language Learning for People Living with Dementia (PLWD)

Recently, the idea of delivering language courses to healthy older adults and people living with dementia (henceforth PLWD) has been put into practice by the Scottish social enterprise Lingo Flamingo [46]. However, already in preliminary discussions with PLWD, carers, health professionals, and residential home staff, it became apparent that the enthusiasm and high expectations associated with such a project were also accompanied by doubts about its feasibility and an understandable worry about potential negative effects of exposing PLWD to learning a new language. While such reservations might reflect the justified concerns about PLWD’s wellbeing, they can also constitute a barrier for the introduction of innovative practices, which might, in the long run, benefit PLWD.

Based on the above-mentioned discussions, two main types of concerns have been raised regarding the introduction of language courses into dementia day-care centres and nursing homes. The first type concerns the possibility that confronting PLWD with new, intellectually demanding material could cause confusion or even agitation, with potential negative effects also affecting other PLWD. The second is that PLWD, unable to achieve the desired success in language learning, might develop frustration and resignation, undermining their self-esteem (often already weakened by the diagnosis) and making them less likely to participate in other activities.

Accordingly, before it is possible to recommend language courses as a non-pharmacological intervention for PLWD (and before we move into the stage of larger randomised-control trials to prove their efficacy), it is necessary to establish whether such an intervention is feasible and well tolerated (while the term *tolerance* is often used in pharmacological trials, here we use the term to refer to the participants’ ability to take part in the course and its activities, without reports of negative side effects, such as agitation). Moreover, it is important to determine how it is perceived not only by PLWD themselves but also by family carers and health professionals.

### 1.3. The Present Study

This present paper addresses the aforementioned questions and aims to assess the feasibility of foreign language learning for people living with early dementia and their family carers, using a qualitative methodology to gather participants’ opinions before and after a language course. To this end, we organised brief (2-week) foreign language courses for PLWD and their carers, delivered by Lingo Flamingo, and we carried out focus group meetings (pre- and post-course) to examine the expectations before the course and its perception afterwards. The language delivered in the two centres reported in this study was Italian—a language popular in the UK with learners of all ages, associated with holidays and popular Italian food, which can be sampled in many Italian restaurants and cafes. Italians were among the first immigrant groups to the West of Scotland (where the courses took place) in the 19th century and still form a sizeable community there.

We also conducted interviews with locality managers at the dementia resource centres where the courses were conducted. We paid particular attention to the possible reporting of any potential adverse effects that could have arisen during or following the courses. Finally, we also wanted to establish the optimal form, frequency, and intensity of the course according to what would be perceived by PLWD, family carers, and managers.

A distinctive feature of our approach was to include in our courses not only PLWD but also their family carers. The idea of joint language courses for PLWD and their carers was suggested to us by several carers during a Dementia Awareness Week conference in Edinburgh in June 2016. Apart from the practical advantages of assisting PLWD in their learning process, we hoped that their inclusion in the courses would give carers a stronger sense of agency and an opportunity to engage with PLWD in a joint activity, allowing them, at least for the time of the course, to change the asymmetrical PWLD–carer relationship into a more symmetrical partnership in learning. Importantly, this study also considered contextual variables supporting the effective delivery of future interventions [47].

## 2. Method

### 2.1. Recruitment of Centres Where the Courses Were Held

In coordination with Lingo Flamingo and Alzheimer Scotland’s (AS) dementia centre managers, Dementia Resource Centres (DRCs) that could potentially take part in the study were identified and contacted. This led to an initial expression of interest received from five AS DRCs. Of this, one centre did not respond to follow-up communications. In the other two locations, the usual weekly schedule of the centre, with the programme and managers changing every day, made it very difficult to accommodate a twice-weekly course, ultimately leaving two AS DRCs (Helensburgh and Kilmarnock) where we conducted the courses reported in this manuscript. In addition, as part of this project, two additional Spanish courses were also carried out in two community centres that were not AS DRCs; one location was sourced through one of the AS DRC Directors, where a Spanish course was carried out at the premises of a community centre in Auchinleck, and only a post-course interview with the community activity organiser but no focus group data were obtained. The second location, in Edinburgh, was outsourced via Lingo Flamingo, and a Spanish course was carried out at a Community Dementia Centre, where a post-course interview with the centre’s dementia support coordinator and pre- and post-course focus groups data were obtained, but only one PWLD-carer pair took part in the focus groups, rendering the focus group session too small. To present results of two more homogenous courses carried out under similar settings and with the same language of instruction, for comparability purposes, we thus only include the AS DRCs Helensburgh and Kilmarnock in this manuscript.

### 2.2. Language Courses

Each course consisted of a total of four 90 min Italian language classes, split over two weeks into two lessons per week. In addition to the classes, there was assigned homework each week (e.g., listening to an Italian song, reading over a short text, going over some of the vocabulary that had been learnt in class or a short role-play scenario with their carer/partner), estimated to require an additional 120 min a week, resulting in approximately five hours of foreign language learning a week. The tutors were freelance Italian teachers who were selected and provided with tailored training by Lingo Flamingo before the classes started. The training included guidelines about teaching and engaging with older adults and how to make language learning accessible for PLWD. The materials used for the classes were developed by Lingo Flamingo, incorporating well-versed dementia-friendly techniques such as sensory learning, music therapy, storytelling, and reminiscing to help create an organic and relaxed learning atmosphere for the learners. As noted, the courses reported in this study took place in Alzheimer Scotland’s (AS) Dementia Resource Centres (DRCs).

### 2.3. Participants

PLWD and their carers were recruited directly by AS DRCs. All were native speakers of English. All of the PLWD and the carers involved in the research gave their consent, and the study was approved by the University of Edinburgh’s Psychology Research Ethics Committee.

Across the two centres, a total of 15 participants (carers and PLWD) took part in the first focus group (FG) session, and a total of 12 participants (carers and PLWD) took part in the second FG session. It was not possible to have the same set of participants in the pre-course and post-course FG sessions; therefore, not all participants present in the first FG session also attended the second FG and vice versa (please see Table 1). In addition, not all participants who were present in the first FG session completed the language course (see drop out reasons in the results section) and, therefore, were not in the second FG; conversely, not all of those who attended the courses had been in the first FG session. In total, six PLWD and two carers were present both at the first (pre-course) and second FG (post-course) (Table 1). An additional couple who attended the course were present at the FG 2 session in Kilmarnock; however, they did not take part in the research and, therefore, were not included in the present analyses. Likewise, a nursing staff member was present in Helensburgh during the second FG session but was not included in the analyses.

### 2.4. Procedure

#### 2.4.1. Focus Groups and Interviews

A total of four focus group (FG) sessions with PLWD and carers were carried out, two in each centre (one before and one after the language course in each centre). In one of the locations, the first FG was carried out one week before the start of the language course; in the other location, it was carried out the day before. The post-course FG sessions took place immediately after the last class of the course in both centres. The setting of the FGs was informal, with nibbles/lunch or tea/coffee. Locality managers were present, but their interventions were kept at a minimum. Brief interviews with the DRC locality managers were carried out after the post-course focus group meetings and subsequently via telephone, approximately one year later as a follow-up.

#### 2.4.2. Qualitative Data Analysis

The FGs and interviews were audio-recorded and transcribed by one of the authors (BB). A content analysis of theme identification from pre- and post-course FGs and interviews after the course was then performed, and relevant themes (see Table 2) were coded using NVivo 11 [48]. In Table 2, items 1 through 5 reflect themes from the questions posed in the FGs, and items 6 through 12 arose in the FGs unexpectedly. Information that was not relevant to the identified themes or from speakers who were at the FG session but were not part of the study was not coded. Theme summaries are presented in the following section.

## 3. Results

In the following sections, we summarise opinions gathered at focus groups meetings by category themes. Participants’ quotes are shown within quotation marks and with the italic typeface. In the transcriptions of quotes*,* you will notice repetition, this is due to transcribing verbatim, including repetition of words and interjections such as “um”, “erhm”, etc. Summaries of the distribution of quotes presented throughout this manuscript are presented in Appendix A (Table A1: quotes by location, participant type, and session; Table A2: quotes by participant).

### 3.1. Thoughts and Knowledge about LL and Previous Experience with LL

#### 3.1.1. Pre-Course

Among the participants (both PLWD and carers) who reported having previous experience with language learning, the languages mentioned included French, German, and/or Latin at school and having used the foreign language on holidays where applicable. One couple reported limited knowledge of/exposure to Italian from holidays.

*PLWD.* Regarding the attitudes towards languages, language learning was perceived by PLWD as challenging: “*I’ve had a shot at various languages and I’ve never been very good at any of them to be honest (MN)*”. However, there was also enthusiasm about learning a new language: “*I’m happy to learn something new (ML)*”. When asked what they knew about the effects of language learning, some of the PLWD and carers were familiar with the idea of language learning as a cognitively stimulating activity: “*We have read a bit in the press about learning a language is very good for people with Alzheimer’s (MN)*” and “*I think it keeps you active a bit, up here* [pointing to head] *(NY)*”.

*Family Carers.* One participant who had been taught French and Latin at school “*found it really difficult to learn a foreign language (NN)*”. The same participant emphasised the difference between school experiences versus learning as fun “*and sometimes you learn something easier when it’s fun […] Whereas when we were at the school it was ‘you’ll do this’ and ‘you’ll do that’ (NN)*”. However, other carers also stressed the elements of challenge and even potential confusion:

“*The initial reaction is that it must be a challenge to someone trying to learn a new language, where they are having difficulty with language at present. […] I simply view it as if it is a program which improves the quality of life of the individual concerned, then it must be a very positive thing. If, on the other hand, it ends up confusing them or adding to their confusion that people are feeling, that might not be such a positive outcome (MT).*”

The reported challenge was perceived to be from both the learners and the teachers: “*It is a challenge on both sides. It goes on the side of people trying to learn, and I suggest it is also a challenge on the part of the person or people who are trying to uhh convey or teach the language (MT)*”.

#### 3.1.2. Post-Course

*DRC Managers.* The idea of language learning as a cognitively stimulating activity was echoed by one of the managers as well: “*I believe in the brain as a muscle that needs to be flexed and this is a very very adept and agile way of flexing […] I also, ehrm, get the ehh idea that umm people that sometimes for people with dementia learning a different language takes some of the pressure off from the issues that they may have recalling their own mother tongue (IR)*”.

We will now turn into the participants’ perceptions about the language course in terms of expectations (pre-course) and level of satisfaction (post-course), including their assessment of course delivery, materials, teaching, and course duration.

### 3.2. Overall Course Perception

#### 3.2.1. Pre-Course—Expectations

*PLWD.* PLWD approached the course with the realisation that it would be a challenge, but also with hopes that it might have a positive effect against dementia: “*I am hoping that anything that I do here, is going to hold back the tide just a little bit (ML)*”; “*probably, hoping it will help continue as I am longer… you are told that it’s going to get worse as time goes on…anything that helps, is a good idea (NY)*”; “*I am quite looking forward to it. The challenge (ND)*”;

“*What we are doing here, but coming here? Well to learn to umm keep understanding words and say them, rather than umm forgetting and ignoring it. So that’s one of my problems anyway. I am not efficient enough at eh, and that’s what causes it, this ehh d- d- dementia, I think. […] Yes, I would like to be better at that *[word recall and production]*, bring it. I mean it was obviously okay a few years okay, but umm when I think of it, it could get worse (LY).*”

One participant also expressed uncertainty about what family members may think of them taking the course: “*And you know, I don’t want to, I’m trying to tell my son. You know, I call him and and he doesn’t know I’m doing this? […] Because he would make a big fuss and uhh send me you know to get get better, read your books and you know, do it (LY)*”.

*Family Carers.* The carers often reported hope that language learning could help the PLWD they care for and mentioned different ways in which this potentially could happen.

“*I think it’s all about enjoyment and quality of life and finding new things that you are comfortable with that also might help keep everything working. […] So, from my point of view, from trying to help dad I think this is, you know, if I can aid that in anyway, that is a good thing. That would be a very positive outcome for me, I think (AY).*”

Importantly, in the context of our joint PWLD-carer design, some carers expressed the hope that the course might be a good experience also for themselves: “*I thought well, somebody told me, they had been involved in this before, and it’s good fun […] I thought, well, even from our point of view, it would be good, you know, and learning something (EN)*”. EN also mentioned they would like to travel to Italy, saying “*I have never been to Italy […] We never just got around to it, but I would like to go, you know (EN)*”, while another carer described they wished to refresh their interest in the language, stating, “*for me, umm, to recharge my interest in Italian. Which I gave up (TN)*”. Another carer reported that they expected that attending the course could improve communication of the PLWD they care for.

#### 3.2.2. Post-Course—Satisfaction

*PLWD.* The general perception of the course was consistently positive, although it did not necessarily correspond to a success in language learning: “*I thought it was very good (JN)”, “very good (SS, ML)*”, “*it was terrific…this has been a bit of fun, hasn’t it? (LY)*”, “*I think it’s been great fun. It’s been very interesting (MN)*”, “*It’s a lovely place, we have been involved in something that has been very enjoyable for all of us (ML)*”, “*I’ve enjoyed the, yes, I’ve enjoyed it. I don’t think I’ve made much progress, but I’ve enjoyed being here (ND)*”.

*Family Carers.* Similar to the PLWD experience, carers also rated the course positively, describing it as “*very good (AS, TN)*”, “*good fun (EN)*”, and “*enjoyable (EN, TN)*”.

*DRC Managers.* The managers also reflected positively on the course: “*I think it went really well. Yeah. I think, umm, as the, as the lessons went on, obviously people relaxed. I think people were really anxious before we started and at the first focus group (TC)*”; the manager also recognised the commitment and efforts of the participants throughout the class project, stating, “*the others that came, I, I think, I’m really proud of them actually, how they embraced the opportunity. And you know, they seemed to be really interested in trying as hard as they could to do something different (TC)*”.

### 3.3. Learning Materials and Homework/Tutor and Teaching Style

#### 3.3.1. Pre-Course

*PLWD.* PLWD wondered whether the course would be comparable to their previous language learning experience at school, as expressed by some of them in the following way: “*Are there, are there…Is there homework that you expect to have? (LY)*” or “*And is it going to be from book learning, or, or what what media? (ND)*”, “*[…] rather than learning verbs (NY)*”. Indeed, when imaging the preferred style for the course, most PWLD expressed the hope that the course would be focused on conversation (IN and NE strongly agreed to the suggestion of a conversationally focused course made by a family carer, TN, in the focus group).

*Family Carers.* Carers similarly expressed the hope that the classes would be more conversational, rather than rote memorisation characteristic of language learning experiences in school: “*we don’t need, we don’t need the written and the reading. As long we get conversation and we can speak to people (NN)*” and “*Conversational […] You know, like you teach a baby. A glass* [pointing to a glass] *(TN)*”.

#### 3.3.2. Post-Course

*PLWD.* Participants were positive about the Lingo Flamingo workbook and appreciated that they could take it home with them, which was perceived positively as constituting a further accessible element of support and practice outside the classroom, e.g., “*You can have it all with you. I mean you can take it home, this book (LY)*”, “*this is a very high-quality resource material. The workbook has been very very well put together, and it will, it will keep up going after we walk out the door (ML)*”, and “*Prefer that* [the workbook] *over the computer (MN)*”. The fact that the workbook was designed with an older population in mind was noted and appreciated:

“*First of all, at a very basic level, the size of the font that has been used is good for people of our age. […] there’s nothing more, em, off putting than getting a dense workbook and it’s all in size 8 font […] the fact that you’ve got a book you’re working through… it focuses you on what you were doing. And you can take, if you were looking at something, you’re like, ‘oh yeah, I remember the week we were doing that’, and you can take that bit out and have a look at it. So, I think the way in which the way the materials we are using have been assembled has been well thought out (ML).*”

Other participants echoed these sentiments. Additionally, the homework was perceived as helpful:

“*I tend to look at things once I’m home just to remember the context, which is recent. And that seemed to work reasonably well. So, I think the, the balance between the amount of, eh, revision that was required and the information we were given, eh, was, was good (ML).*”

PLWD also appreciated the activities employed: “*I thought the activities were well chosen. Variety (ML)*”.

*Family Carers.* The carers also found the workbook useful as a support tool and for revision, stating, “*the book was a sort of back-up […] Which was good to go over it again. That, that is a good thing (AN)*”, “*I found it helpful (EN)”*, “*It is good to keep track, look it up […] You could look it up in the book (TN)*”, and they liked it in terms of content, saying, “*I think it was written quite clearly and it was interesting (AN)*”. The only suggestion to improve the materials (workbook in particular) was “*I felt it jumped about a bit […] And then there was something entirely different. It didn’t connect to the thing you were just doing […] You know, it sort of leaped (TN)*”.

The carers had positive comments on the tutors’ teaching style, saying, “*She didn’t make you feel ’oh, you’re thick’, you know? She just, she was good (AN)*”, “*she was very good. She made it good fun, she really did. And I think you learn better, it was enjoyable (EN)*”, and “[she] *came across more interesting than I thought it would be. I thought it would be quite mundane. […] she’s a lively wee bird […] She engaged everybody, that’s probably it (TN)*”.

*DRC Managers.* The teaching materials were described as “accessible”, and the workbook seemed to have been a useful tool to practice, in particular for PLWD, as reported by one of the managers, “[PLWD’s name] *I think has got half the book done (TC)*”. The enthusiasm about using the materials beyond the classroom was also observed when a manager reported that a participant who missed a class asked the tutor for work to do at home.

Managers appreciated that the lessons were well suited to the population and delivered in a pace that did not impose pressure on the participants, stating that

“*[…] the first impression of what is about to happen is not strict, it is not draconian, it is not ehh, it’s not going to place undue pressure on them… it’s not like a classroom […] It’s not, ’right, today we are doing six verbs and you have to learn them’ (IR).*”

and

“*Everybody was learning the same…one of the best things, *[tutor’s name] *I thought was brilliant, but one of the best things was that if people weren’t quite getting something, she didn’t labour it. You know, if there was something that, you know, was proving to be tricky, then we moved on. So, we did it for a little bit but we didn’t, there was no labouring it, there was no, people didn’t feel, I didn’t think they were made to look like they were struggling (TC).*”

Managers also appreciated the variety of the activities, saying,

“*[…] I think also, because of the enthusiasm and the variety of themes and topics explored, umm, you know, if if you are getting tired of the words and the language, it’s not long before you get another different stimulus coming up in terms of the music and the pictures (IR)*”,

and

*She did show a piece of film one day, yes. It was good, that variety, I think they liked that.* “*[…] When, umm, attention span is short, moving, you know, onto the different things, I think that was very helpful (TC).*”

### 3.4. Course Duration and Frequency

#### 3.4.1. Pre-Course

*PLWD.* When asked about the ideal length and frequency of the course (if they were going to “design” their own course), one participant suggested “*just once I would think (IN)*” [a week], and the participants in this group also recognised that different participants might have different needs and preferences: “*I suppose that we might all go at different paces […] That’s a bit difficult, at least until we see what we are actually going to do. And how we cope with it (NY)*”, “*well, that depends on the demands that are made on you (ML)*”, “*I don’t know, I would say fair. I don’t know, just take it as it comes and see how it goes, see how it develops (MN)*”.

*Family Carers.* About the ideal length and duration, one carer said, “*a few times a week probably (EN)*”, others said “*maybe a couple of times a week (NN)*”, and one carer did not show a concrete preference “*I don’t know (TN)*”.

#### 3.4.2. Post-Course

*PLWD.* PLWD generally found the frequency of the sessions (90 min twice per week) appropriate but would not suggest doing more than that:

“*I don’t know, on the fence, once or twice a week. I think it was a reasonable time and duration. Eh, I would quite like it to be a wee bit longer, but I tend to get stressed a wee bit. I get tired (MN).*”

The duration and frequency were also described as “*comfortable (ND)*”, “*the time was fine I think (JN)*”, “*Well not more than that (LY)*”, and “*Language learning needs continuity, and to do anything less than two I think would be hampering our progress (ML)*”.

*Family Carers.* The carers found the duration and frequency appropriate, stating, “*that was just right […] Two a week is maybe enough, for us anyway (EN)*” but would have liked the course to continue for longer “*not more in the one week, but carrying on from here I would prefer it going for week after week after week. Like say like a school term (TN)*”. The same carer made a suggestion to have the classes less frequently so that it could go for longer: “*Instead of having it a a crunched-up thing like this, having it once a week but an extended number of weeks, for Italian (TN)*”.

*DRC Managers.* Regarding the duration of the class, initial doubts were expressed by one of the managers, who stated,

“*at the outset, I thought an hour and a half is quite a long time. And I wasn’t sure how people would be with a lesson at an hour and a half. But it, now I think people were tired afterwards, they said they were tired afterwards, but they participated right through, there wasn’t really any flagging, I didn’t feel […] And the time did seem to go quickly (TC).*”

A hypothetical schedule of three hours spread over three days per week was, however, perceived by the manager as not feasible: “*I think maybe, ehm, in terms of concentration maybe the three hours. But I don’t think we would have gotten people three times in a week (TC)*”.

The following section delves into the participants’ perceptions of social aspects.

### 3.5. Social Interaction

#### 3.5.1. Pre-Course

*PLWD.* Interestingly, course-related expectations were not confined to potential cognitive benefits but extended to social interaction: “*I live alone, I and ah, umm I think that’s what brought this vacant kind of idea, that I can’t make myself. You know, because I don’t have any reason to speak. So, I’m trying, you know, that’s why I’m here. To try and mingle and learn a bit more (LY)*”.

*Family Carers.* No specific expectations were expressed in terms of social interactions by carers in the first focus group.

#### 3.5.2. Post-Course

*PLWD.* An important observation mentioned by some participants in this group was the sense of socialising: “*Aye, I thoroughly enjoyed it…Got a lot of good laughs out of it, it was good (JN)*”. A participant who, in the pre-course focus group, referred to living alone, mentioned that [as a group] “*we have to stick together (LY)*”; another participant commented “*highlights were the comradery […] the surroundings are, are bright and cheery […] not only in the surroundings, but also with the people who are here (ML)*”.

*DRC Managers.* Speaking for the course carried out for this study, as well as previous experiences with Lingo Flamingo courses, social aspects and sense of bonding were expressed by one of the DRC managers:

“*I think that’s that’s probably if, you know if not as powerful, if not the most powerful element of these courses is that we have, this is the third time we’ve brought together individuals that have never met one another before. They have bonded through laughter, they have bounded through the the shared attempts to learn a language. Ehrm they have a lot of common ground to discuss afterwards at the lunch. There’s, you know, there’s a number of carers going through the same thing as another. So there’s a, there’s a natural support function that comes umm during the courses and after the courses. And friendships, ehh, actually are formed (IR).*”

Another manager referred that even though no one knew each other at the beginning of the course,

“*they relaxed with each other, and they relaxed with us. I didn’t know everybody that was in the group, before they came…I think they obviously, they said they felt comfortable, but they appeared to feel comfortable because their behaviour changed and that way they interacted changed as well. And really in quite a short time, it’s only been a few weeks. So yeah, they’ve kind of come together (TC).*”

The manager even raised a point about companionship as an effect of the class:

“*And somebody had made a point, and I’d heard this a few times, about ’we are all in it together’. So, I think, I think that’s something people kind of felt ’oh I might be finding it hard, but I know everyone is the same, and we are all, kind of, yeah’ I think [name] made mention of that, about the company and the companionship. I think maybe a couple of them did. I mean you can see, I think you can see that (TC).*”

### 3.6. Stress

#### Post-Course

*PLWD.* PLWD did not report feeling pressure in the class, stating,

“*there’s no sense of pressure or anything like that. It’s a relaxing environment, and we’re all starting from the same point,* i.e., *nothing. […] This is a bright, comfortable environment. The people were working with, umm, both on this side of the table and that side of the table are absolutely as one, eh, no one is trying to score points or make you feel that you’re under pressure in anyway, eh, which sometimes happens in learning (ML).*”

One participant reported that “*It was good because I was, you know, stopping and not doing anything. And when you get a wee bit of fun like that, you relax. And you start to do a bit better, you know (MN)*” and, importantly, that “*It was much more light-hearted than I thought it would be (MN)*”, thus indicating that the course left a positive impression.

*Family Carers.* The carers also agreed that the course had not been stressful: “*I am quite happy with the way the course was delivered. ‘Cause it, we already said, it was no stress (EN)”*, “*it wasn’t stressful (TN)*”, and moreover, EN also said, “*And I think you learn better, when you are not pressured. No pressure (EN)*”.

*DRC Managers.* A manager said the method of teaching allows participants to relax: “*I think you relax, you relax into that method of learning (IR)*”. Another manager mentioned that in spite of the potential stress that one could experience before the start of the course (because people might make associations to traditional school or to the anxiety of starting something new),

“*I think as well as the fear of, emm, learning a language in school was really stressful, there is the actually, doing most things is really stressful and this is something new. So, this could be even more stressful. So, I think that’s, you know, maybe part of the anxiety that people have before they come. And yet, they came so, I’m so admiring of them. Because I think it is difficult for people to come (TC).*”

Importantly, neither PLWD, carers, nor managers reported cases of confusion, agitation, or other negative reactions.

### 3.7. Tutor versus Computer

#### Post-Course

*PLWD.* There was agreement among the PLWD who gave their opinion that it was an advantage to have the course taught by a “real person” rather than via a computer/app: “*it* [the class] *was terrific. I just feel that we’ve got the real, the real eh sound of it. You know, to get the real, a person that comes and was Italian, or is Italian. And we can learn from that (LY)*”. One participant reported not being “*very fond of computers (ML)*”; therefore, a sense of appreciation of having the in-person interaction with the tutor was gathered.

*DRC Managers.* The advantages of having a “real” person tutor versus a computer included the emotional aspect and facial expressions—“*[…] actually, what those individuals that are participating enjoy, is ehh reading the the facial messages that is coming from everybody else that is participating as well as it being much easier for the tutor to become that kind of focal point and that that vibrancy (IR)*”.

### 3.8. Most Difficult Aspects of the Course

#### Post-Course

*PLWD.* Among aspects they may have found difficult in class, one PLWD mentioned that “*I have problems with syntax sometimes (MN)*”.

*Family Carers.* Once prompted to describe what parts they found difficult, only one carer replied that “*Sometimes I thought it was a bit too much, to me anyway […] But it was, I enjoyed (AS)*”.

*DRC Managers.* Finally, when inquired whether there were difficult bits in the class (for the participants), in line with reports from PLWD themselves, who mentioned *syntax* as being something difficult for them, this manager also referred that they noticed difficulties in putting things together “*Some of it I thought was absolutely right. I think they all struggled a little bit with the verb […] The verb part. It was making a sentence, wasn’t it? (TC)*” and gave more particular examples:

“[PLWD’s name] *found that very difficult too, she needed quite a bit of one-to-one support to manage that. She did *[manage it]*, but she needed quite a bit. So that’s quite interesting. That’s the sequencing, the putting together. […] it was putting them together, two things to make a sentence. […] So, it was, it wasn’t the phrases. Cause they had all the phrases independently. But the matching, that seemed to be tricky (TC).*”

### 3.9. Logistics

#### Post-Course

*PLWD.* Only one PLWD mentioned a transportation issue, namely, to have had to walk a long distance because of living on their own without a car, “*because I live up at the top of the hill, and I don’t have a car, my time spent, I spend an awful lot of time walking up and down the hill (ND)*”.

*DRC Managers.* One of the managers reported that some participants declined the initial invitation to take part in the study during the recruitment phase (exact number not provided). Citing reasons why, according to the manager, “*the carer in that scenario became, couldn’t be convinced that it wasn’t something that would put their loved one at some degree of either embarrassment, discomfort, or umm feelings of inadequacy (IR)*”.

### 3.10. Other Outcomes: Memories and Achievement

#### Post-Course

##### PLWD

*Memories from the past.* One of the outcomes noticed by the participants was that learning the language brought back memories either from life-events such as trips and holidays “*when we went to Italy and quite a lot of the things that we actually saw when we were there, was actually show again when we were here (JN)*”. Indeed, ND recounted that

“*I was sitting at home, and I opened up the book, and I just saw the map on one side, and it was the map of Italy. And, umm, I thought, I could, I, it, it, I felt I could, it had a memory. But I couldn’t bring it forward into my brain. So, I carried on just looking at the other pages, and then it suddenly clicked into me that this map of Italy was where my husband and I had been, umm, we had six holidays in Italy and abroad. And I had totally forgotten them (ND).*”

The same participant reported having had a similar experience looking at a map of Norway that also brought back additional memories, thus suggesting that such reminiscing was not only associated with the country of the language they were learning. Within this theme, the opinion of two PLWD in session 2 could be linked to what they had said in the session before the course.

Among the memories brought back were those of foreign languages learnt previously in life—for example, in the case of a PLWD who, in session 1, referred not have been good at learning languages in the past now reported, “*[…] I tend to use German quite a bit, whereas I never used to do. And in fact, when I was in school I think I got 13 out of 100 for German. […] Yeah, I can remember, I can remember, oh, poems and things in German. Yeah (MN)*”. This same participant even reported a positive side-effect beyond the cognitive and at the physical level, linking it to the experience of having attended the course: “*I think my fingers work a lot better than they used to. But who knows. It’s been really good (MN)*.”

*Sense of achievement.* Another outcome of the courses was a positive effect on PLWD’s self-confidence. This was expressed by a participant who had reservations about telling their son about the course, and in the post-course session exhibited a sense of achievement: “*my children are coming, […] so, I’m hoping that I can use it, you know, and show them, how to speak English, or French I mean, no! Italian (LY)*”.

*DRC Managers.* Attending the language courses may offer the possibility for participants to attend other activities offered by the centres, as noted by one of the managers:

“*From our perspective what it means, what it meant for the last group and definitely what it will mean for this group, is that we can then introduce other therapies and interventions to exactly that cohort […] it further enhances our our centre and it further enhances exactly why this building is here. Umm and and the mantra of people not living alone is fulfilled (IR).*”

The manager also commented that the carers themselves could also benefit from the courses: “*[…] you also as a carer, and maybe unbeknownst to them, get a chance to flex that muscle and prevent as well as (IR)*”. Another secondary effect of the courses was that learning a foreign language could make the participants more likely to travel and be immersed in the culture of the language they learnt: “*And I think, you know, ehh, when you are learning a foreign language, most of the time, you’re learning it so that when you go to Italy, I think you and your wife are going to Italy next year* [direct at participant] *(IR)*”. Another manager expressed how initially some participants new to the centre were unsure that they would be able to do well in a language class. By the end of the course, a positive effect on PLWD’s self-esteem and confidence was also expressed by one of the managers: “*I think it was really good for people’s self-esteem and for their confidence once they come. Once they come and they try it and they realise, actually ’I’m doing just as well as anybody else here’ (TC)*” and even a sense of pride, reinforcing the observation that one of the participants was even keen to tell her family members about having taken part in the language course. In line with PLWD’s reports, one manager provided further support to the outcome that the language course produced a retrieval of some memories in the participants, speaking about one particular PLWD: “*the Italian words and names had triggered memories that she* [the PLWD] *thought had been completely lost…what she actually said to me was ’I had a lovely evening last night, sitting and reminiscing about lovely times I had with my husband’ (TC)*”.

The manager also commented that the carers themselves could also benefit from the courses: “*[…] you also as a carer, and maybe unbeknownst to them, get a chance to flex that muscle and prevent as well as (IR)*”. Another secondary effect of the courses was that learning a foreign language could make the participants more likely to travel and be immersed in the culture of the language they learnt. “*And I think, you know, ehh, when you are learning a foreign language, most of the time, you’re learning it so that when you go to Italy, I think you and your wife are going to Italy next year* [direct at participant] *(IR)*”. Another manager expressed how initially some participants new to the centre were unsure that they would be able to do well in a language class. By the end of the course, a positive effect on PLWD’s self-esteem and confidence was also expressed by one of the managers: “*I think it was really good for people’s self-esteem and for their confidence once they come. Once they come and they try it and they realise, actually ’I’m doing just as well as anybody else here’ (TC)*” and even a sense of pride, reinforcing the observation that one of the participants was even keen to tell her family members about having taken part in the language course. In line with PLWD’s reports, one manager provided further support to the outcome that the language course produced a retrieval of some memories in the participants, speaking about one particular PLWD: “*the Italian words and names had triggered memories that she* [the PLWD] *thought had been completely lost…what she actually said to me was ’I had a lovely evening last night, sitting and reminiscing about lovely times I had with my husband’ (TC).*”

In the following section, we present a summary of suggestions received from participants regarding future improvements to the course.

### 3.11. Suggestions

#### Post-Course

*PLWD.* When asked about suggestions on how to improve the course, one participant said, “*there is nothing that I would change about it. […] next time you’re doing it, do it again, the same way (ML)*”. The general impression was that the courses were good, with no changes needed.

*Family Carers.* Carers suggested to have a recap of what had been covered in previous classes at the beginning of each class, stating, “*A recap and then go on? […] That might, you might remember it better that way (AN)*”, “*Yes, you keep getting it all with the same and a wee bit more added on you remember it better that way (AS)*”, and “*If you are getting it over and over again, it would sink in better (EN)*”.

*DRC Managers.* One of the managers considered that having food or a dining setting is important, as this helps to bring people together. Due to the nature of this project, which was aimed at exploring participants’ ideas before the course (uninfluenced by the experience of the course itself), there was no taster session; one manager suggested that if they were to do a course again, they would have a taster session.

### 3.12. Drop out Reasons

#### Post-Course

*DRC Managers.* Reasons for participants to drop out during the project were as follows: a PLWD was not feeling well physically; therefore, both the PLWD and carer did not manage to attend any longer. One PLWD stopped attending classes (recorded in researcher’s notes). Another PLWD stopped participating in the study after the first focus group because of unease regarding speaking in public, and the carer dropped out as well; therefore, neither of them attended the language course. Finally, another participant, according to the manager (TC), seemed to do well in the language class—“*he was participating the same as anybody else. And, emm, you know, laughing, and he looked comfortable*”, but “*he felt he wasn’t able to do it properly*”, as reported by the manager, and decided not to continue attending the course. Of note, the same participant had also tried out another activity at the centre of which he had also dropped out. In this case, since the PLWD dropped out of the study, so did their carer.

### 3.13. One-Year Follow-Up Interviews with DRC Managers

The AS DRC locality managers in both centres reported that the PLWD who took part continued to speak of the courses with fondness and retained specific memories: “*people refer to it affectionately. You know, they will talk about, ’When we did the Italian’ and ’Do you remember when we did the course?’ Some people do remember specific things from it (TC).*” The impression was that “*people probably would have continued with it if we continued to provide it. But I think that, you know, for people to go and find a language course somewhere and continue it by themselves is probably a big step for folk (TC)*”. The positive feeling about the courses was also noticeable in the carers and went beyond language learning; “*it enabled carers to see what people with dementia were capable of eh engaging with and also retaining (IR)*”.

Finally, both managers commented on the social effects of the courses, saying, “*the wives of the gentlemen have now created their own little social group (IR)*” and

“*[…] but certainly the things which might be considered spinoff benefits like the friendships and like the support that we observed people giving to each other during the course. I think that those things in themselves gave great value to doing the course… actually I think that, you know, those are probably the things that have been the greatest benefit to people (TC).*”

## 4. Discussion

The aim of this study was to assess the feasibility of foreign language learning for people living with early dementia and their family carers, using a qualitative methodology to gather participants’ opinions before and after a language course.

The experience of language courses for people living with dementia (in early stages) and their carers described in this study, accompanied by focus group meetings, suggest that the procedure is both feasible and well tolerated. The most difficult aspects of the courses were connected to their practical organisation, including fitting into the DRC schedule, finding suitable rooms, or arranging transport.

The initial focus groups, conducted before the beginning of the course, showed, among PLWD, carers, and managers alike, a combination of positive expectations and enthusiasm but also worries, particularly about the risk of confusion and frustration, should the programme prove too difficult for the participants. None of these negative effects were observed at any stage of the courses. The final focus group meetings revealed a consistently positive evaluation of the course.

The courses were described with positive terms such as “enjoyable”, “good fun”, and “terrific”. Although some aspects of the courses were perceived as challenging, the successful overcoming of these difficulties gave PLWD a feeling of achievement and pride, a perception that, according to one of the managers, extended to the carers (“*it enabled carers to see what people with dementia were capable of*”). The follow-up interviews with the managers showed that the courses were still fondly remembered by the participants one year after they had finished.

In terms of course design and implementation, the approach taken by Lingo Flamingo was well received. Participants preferred being taught by “real people” rather than a computerised format. They were also happy with the intensity and frequency of the lessons; two 90 min sessions per week were well tolerated and perceived as stimulating but not exhausting. Participants expressed the wish to continue with similar courses in the future. One of the most striking observed effects was the social bonding, comradeship, and friendship that developed among the participants. People who did not know each other became friends; the common experience of learning a new language (“*we are all in it together*”) created a feeling of community. According to the managers, these social bonds (among PLWD as well as among carers) had lasting effects continuing long after the end of the courses. For PLWD, attending the language course provided a valuable opportunity for social interaction, especially considering the frequently observed social withdrawal in this population [8].

### Limitations and Future Directions

Our study has limitations. First, its exploratory nature limits its scope to participants’ perceptions within a small sample size. The learners in this course were PLWD (in early stages) and their family carers; thus, further research is needed to explore the generalisability of our findings. Second, participants could potentially have felt constrained from offering negative comments in the focus group setting. However, we tried to minimise this risk by conducting the focus groups in a comfortable environment. Also, it has been argued that participants may feel more comfortable disclosing negative comments in group discussion settings [49]. We also complemented the focus groups with interviews with the centres’ managers. Third, in addition to the small sample size, the short duration of the courses allows us to draw conclusions only regarding the feasibility of delivering such courses, which was the overarching aim of this study, but it is important to emphasise the need to conduct studies on the effectiveness of delivering such courses by using randomised controlled trials with larger sample sizes and longer-duration courses. In particular, the idea of learning an additional language in later life has been proposed as cognitive therapy for age-related decline [19,20]; however, less is known about the effects of language learning on psychosocial aspects in people already living with dementia, as well as on their family carers. Our study shows that tailored language teaching for this population is not only possible, but it can also offer benefits (e.g., social interaction, sense of achievement, bringing memories from the past), opening an avenue for exploring this area and paving the way for informing future interventions assessing the effectiveness of such courses in future randomised controlled trials.

Nevertheless, while the participants in our study reported positive social aspects of attending the course, future interventions could focus on assessing the psychosocial effects of language learning in this population. If effective, language learning courses have the potential to be considered as an option among other non-pharmacological activities aimed at increasing opportunities for social interaction for PLWD, such as those reviewed in [13].

Furthermore, identifying the optimal frequency, intensity, and duration of language courses for this population in future interventions is crucial to minimizing attrition and making it manageable for PLWD and their carers. In this vein, within the limited literature on the effects of language learning on psychosocial aspects, the pilot study conducted by Pfenninger and Polz [45] showed improvements on psychosocial aspects after an intensive 4-week language course. The course included three lessons of two hours per week, but importantly, their study focused on a population of healthy older adults. Of note, in the study by Bak et al. [28], the improvement in attentional switching associated with an intensive language course remained in those who continued practicing the language, training for at least 5 h a week. However, the study was again conducted on healthy adults, with a wide age range (18–78) and was focused on cognition (attentional functions). In sum, the development of future language interventions for PLWD should determine the optimal duration, frequency, and intensity with the population in mind.

## 5. Conclusions

Summarising, our study demonstrates that foreign language courses are feasible to administer and well tolerated in people living with early dementia. No negative effects, such as confusion or agitation, were reported. On the contrary, the courses were perceived as enjoyable and gave PLWD a sense of achievement and pride. Using accessible materials and tailored lessons, and once practical obstacles such as scheduling or arranging transportation are overcome, the courses can be well integrated into the routine of a dementia day-care centre and even have the potential to increase attendance of other activities. The inclusion of carers can bring multiple benefits for them personally, as well as for their relationship with PLWD and their interaction with day-care centres and their teams. Finally, the impressive and lasting social effects (as confirmed by our one-year follow-up interviews) could be particularly relevant in times in which loneliness and social isolation are increasingly perceived as a particularly problematic aspect for later adulthood and people living with dementia.

## Figures and Tables

**Table 1:** Participants’ demographic information and number of participants at each focus group session on each location.

Participant Group	ID	Gender	Age	Location	FG Attendance
PLWD					
	MN	Male	79	Helensburgh	FG 1 and FG 2
	ND	Female	73	Helensburgh	FG 1 and FG 2
	LY	Female	78	Helensburgh	FG 1 and FG 2
	NT	NA	NA	Helensburgh	FG 1
	NY	Male	73	Helensburgh	FG 1
	ML	Male	66	Helensburgh	FG 1 and FG 2
	NE	Male	81	Kilmarnock	FG 1
	JN	Male	74	Kilmarnock	FG 2
	BN	Male	59	Kilmarnock	FG 1
	ON	Male	76	Kilmarnock	FG 1 and FG 2
	IN	Male	81	Kilmarnock	FG 1 and FG 2
	SS	Male	72	Kilmarnock	FG 2
Family Carers					
	MT	NA	NA	Helensburgh	FG 1
	AY	Female	36	Helensburgh	FG 1
	AN	Female	72	Kilmarnock	FG 2
	EN	Female	76	Kilmarnock	FG 1 and FG 2
	TN	Female	80	Kilmarnock	FG 1 and FG 2
	NN	Female	58	Kilmarnock	FG 1
	AS	Female	71	Kilmarnock	FG 2

Note: PLWD = people living with dementia. FG denotes focus group. NA denotes attendance only at FG 1; demographic information not obtained.

**Table 2:** List of themes for the focus groups (FG) in session 1 before the course, session 2 after the course, and in the post-course interview with the Dementia Resource Centre (DRC) managers.

Themes	FG Pre-Course	FG Post-Course	Interview Post-Course (DRC)
1. Thoughts and knowledge about LL and previous experience with LL	**✓**		**✓**
2. Overall course perception (expectations/satisfaction)	**✓**	**✓**	**✓**
3. Learning materials and homework/tutor and teaching style	**✓**	**✓**	**✓**
4. Course duration and frequency	**✓**	**✓**	**✓**
5. Social interaction	**✓**	**✓**	**✓**
6. Stress		**✓**	**✓**
7. Tutor versus computer		**✓**	**✓**
8. Difficult aspects		**✓**	**✓**
9. Logistics		**✓**	**✓**
10. Other outcomes: memories and achievement		**✓**	**✓**
11. Suggestions		**✓**	**✓**
12. Drop out reasons			**✓**

Note: Ticks denote whether the opinions were coded under the listed theme at least at one FG (Helensburgh or Kilmarnock) or at least one of the interviews with DRC managers. LL = language learning.

## Data Availability

The qualitative dataset and transcriptions are not publicly available due to privacy issues.

## Appendix A. Quotes Summaries

**Table A1:** Distribution of the quotes presented in this manuscript by location, participant type, and session.

Location	*n*	Participant Type	*n*	Session	*n*
Helensburgh	66	PLWD	51	S1	29
Kilmarnock	51	Carer	36	S2	83
		Manager	30	1-Year follow-up	5

Notes: *n* = number of quotes; PLWD = people living with dementia; S1 = focus group Session 1 (pre-course), S2 = focus group session 2 (post-course).

**Table A2:** Distribution of the quotes presented in this manuscript by participant.

PLWD	*n*
ML	14
ND	6
MN	11
LY	10
NT	0
NY	4
IN	1
ON	0
NE	0
BN	0
JN	4
SS	1
Total	51
**Carers**	*n*
AY	1
MT	2
EN	11
TN	11
NN	4
AN	4
AS	3
Total	36
**Managers**	*n*
TC	18
IR	12
Total	30

Notes: *n* = number of quotes; PLWD = people living with dementia.
